# Epitope Profiling Reveals the Critical Antigenic Determinants in SARS-CoV-2 RBD-Based Antigen

**DOI:** 10.3389/fimmu.2021.707977

**Published:** 2021-09-21

**Authors:** Min Jiang, Gaiping Zhang, Hongliang Liu, Peiyang Ding, Yunchao Liu, Yuanyuan Tian, Yanwei Wang, Aiping Wang

**Affiliations:** ^1^School of Life Sciences, Zhengzhou University, Zhengzhou, China; ^2^Henan Zhongze Bioengineering Co., Ltd., Zhengzhou, China; ^3^Key Laboratory of Animal Immunology, Henan Academy of Agricultural Sciences, Zhengzhou, China

**Keywords:** SARS-CoV-2, spike protein, RBD, monoclonal antibody, epitope

## Abstract

The ongoing COVID-19 pandemic caused by SARS-CoV-2 is a huge public health crisis for the globe. The receptor-binding domain (RBD) of SARS-CoV-2 spike (S) protein plays a vital role in viral infection and serves as a major target for developing neutralizing antibodies. In this study, the antibody response to the RBD of SARS-CoV-2 S protein was analyzed by a panel of sera from animals immunized with RBD-based antigens and four linear B-cell epitope peptides (R345, R405, R450 and R465) were revealed. The immunogenicity of three immunodominant peptides (R345, R405, R465) was further accessed by peptide immunization in mice, and all of them could induced potent antibody response to SARS-CoV-2 S protein, indicating that the three determinants in the RBD were immunogenic. We further generated and characterized monoclonal antibodies (15G9, 12C10 and 10D2) binding to these epitope peptides, and finely mapped the three immunodominant epitopes using the corresponding antibodies. Neutralization assays showed that all three monoclonal antibodies had neutralization activity. Results from IFA and western blotting showed that 12C10 was a cross-reactive antibody against both of SARS-CoV-2 and SARS-CoV. Results from conservative and structural analysis showed that ^350^VYAWN^354^ was a highly conserved epitope and exposed on the surface of SARS-CoV-2 S trimer, whereas ^473^YQAGSTP^479^ located in the receptor binding motif (RBM) was variable among different SARS-CoV-2 strains. ^407^VRQIAP^412^ was a highly conserved, but cryptic epitope shared between SARS-CoV-2 and SARS-CoV. These findings provide important information for understanding the humoral antibody response to the RBD of SARS-CoV-2 S protein and may facilitate further efforts to design SARS-CoV-2 vaccines and the target of COVID-19 diagnostic.

## Introduction

Common coronaviruses (CoVs) have been circulating in humans for a long time, which usually cause mild to moderate diseases, like the common cold. However, three beta-CoVs (SARS-CoV, MERS-CoV and SARS-CoV-2) infections have caused large outbreaks in recent years (1–3). Especially, SARS-CoV-2 has caused a global pandemic, namely the coronavirus disease in 2019 (COVID-19) (4). According to real-time data from Worldometer (updated on August 12, 2021), 220 countries and territories around the world have reported a total of 205,512,912 confirmed cases of the coronavirus COVID-19 and a death toll of 4,337,588 deaths (https://www.worldometers.info/coronavirus/countries-where-coronavirus-has-spread/). Unfortunately, the first COVID-19 wave has never really ended in some countries, and a new COVID-19 surge is on track this fall and winter, meaning more severe COVID-19 cases and potentially higher mortality (https://www.cdc.gov/coronavirus/2019-ncov/covid-data/covidview/index.html). Various modalities of vaccines against SARS-CoV-2, based on different routes and immunization procedures, have been approved for marketing worldwide (5). However, the antigen epitopes in these vaccines are poorly understood.

Similar to SARS-CoV in genome structure, SARS-CoV-2 has a single-stranded positive-sense (+ss) RNA genome varies from 29.8 kb to 29.9 kb in length, including two large ORFs (ORF1a and ORF1ab) encoding the polyproteins (pp1a, and pp1ab), four structural protein genes encoding proteins envelope (E), membrane (M), nucleocapsid (N) and spike (S), and some accessary protein genes (i.e., ORF3a, ORF6, ORF7a, ORF7b, ORF8, ORF10) (6, 7).The entry of SARS-CoV-2 into its host cells depends on interaction between the S protein with the angiotensin-converting enzyme 2 (ACE2) receptor on host cells and virus-host membrane fusion mediated by S protein (8). As other CoVs, the S protein of SARS-CoV-2 is cleaved into two functional subunits, S1 and S2, *via* the furin site (682-685 aa) (9). Binding with ACE2 triggers membrane fusion activation, in which S is further cleaved by a second proteolytic site (S2′) to release fusion peptide (10, 11). Therefore, hindering viral engagement with ACE2 is an efficient strategy to prevent the virus entry. In addition, the S glycoprotein of CoVs is surface-exposed. Multiple studies have been launched to assess the immunogenicity of structural domains of S protein. Currently, most of the potent antibodies are against CoVs RBD (11–14). This makes the RBD of SARS-CoV-2 S protein is the primary candidate for clinical interventions and vaccine design (15, 16).

The high-resolution structure of SARS-CoV-2 RBD bound with ACE2 suggested that the overall ACE2-binding mode of SARS-CoV-2 is similar to SARS-CoV (17–19). According to amino acid alignment, the RBDs of SARS-CoV and SARS-CoV-2 share 73.5% homology (20). Because of the high similarity in structure and sequence, the RBDs of the two viruses may have cross-reactive epitopes which can induce cross-reactive antibodies. The serum of SARS-CoV convalescent patients and several SARS-CoV antibodies have been shown to confer react to SARS-CoV-2 as well (21–25). However, there is a gap in knowledge on the broad cross-protective epitopes shared between SARS-CoV-2 and SARS-CoV. Currently, findings on SARS-CoV-2 B cell epitopes mainly include the determination of antigen-antibody structural complex, bioinformatics prediction and Pepscan (26–29). Undoubtedly, determination the complex structure is the most accurate method for epitope identification, but it is not readily applicable to many antigens and antibodies, for its laborious efforts with a low success rate. The accuracy of bioinformatics prediction is unclear and the obtained epitopes need further experimental verification. The sera (polyclonal antibodies) from COVID-19 convalescent individuals were mostly used to identify SARS-CoV-2 epitopes by the Pepscan method (30–32). Further studies that involve the determination of the minimum functional motif for antibody binding and the isolation of the monoclonal antibodies (mAbs) targeting these linear epitopes will be needed.

Here, we attempted to analyze the antibody response to the RBD of SARS-CoV-2. We screened the linear B-cell epitope peptides in a panel of sera from animals (swine/mouse) immunized with RBD-based antigens using overlapping peptides spanning the RBD of SARS-CoV-2 S protein. After synthesis and conjugation, the immunogenicity of these immunodominant epitope peptides was further validated by immunizing mice. Furthermore, the monoclonal antibodies binding to these immunodominant epitope peptides were generated and characterized. In addition, the variable regions of these antibodies were sequenced and the immunodominant epitopes in the SARS-CoV-2 RBD were finely mapped. The conservation of these epitopes was analyzed across various virus isolates. The spatial distribution and structural property of these epitopes were analyzed by mapping to the structures of SARS-CoV-2 RBD-ACE2 complex and S trimer.

## Materials and Methods

### Cells and Serum

Human embryonic kidney 293T (HEK293T) cells were obtained from ATCC (Manassas, VA, USA) and maintained in Dulbecco’s modified Eagle’s medium (DMEM, Solarbio, Beijing, China) supplemented with 10% (v/v) fetal bovine serum (FBS, Gibco,USA). The sera of animals used to screen the linear B-cell epitope peptides and SP2/0 myeloma cells were kindly provided by Henan Provincial Key Laboratory of Animal Immunology, Henan Academy of Agricultural Sciences (Zhengzhou, China). These animals were vaccinated with the recombinant proteins designed based on SARS-CoV-2 RBD containing adjuvants (aluminum hydroxide/CpG1018). SP2/0 cells were maintained in Roswell Park Memorial Institute 1640 (RPMI1640, Solarbio, Beijing, China) medium supplemented with 10% (v/v) FBS (Gibco, USA).

### Peptide Design and Synthesis

**Figure 1:**
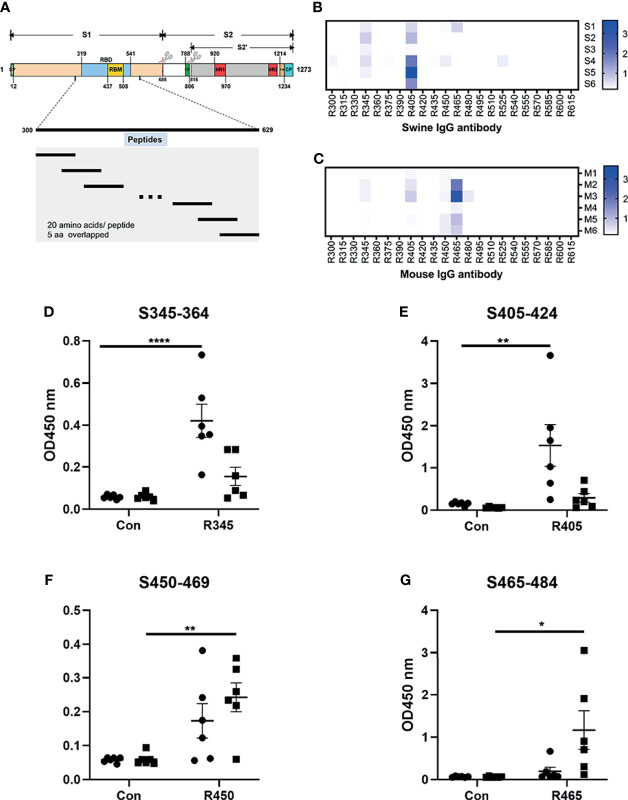
Analysis of B-cell epitope peptides of serum antibodies against SARS-CoV-2 RBD. **(A)** Schematic representation of the functional domains of S protein of SARS-CoV2 and the overlapped peptides spanning the RBD of SARS-CoV2. The S protein consist of two functional subunits (S1 and S2). The S1/S2 cleavage sites are indicated by the scissors. SP, signal peptide. FP, fusion peptide. HR1, heptad repeat 1. HR2, heptad repeat 2. TM, transmembrane domain. CP, cytoplasm domain. RBD, receptor-binding domain. RBM, receptor-binding motif. The amino acid residues number in each domain indicates their position in the S protein. **(B, C)** The reactivity of sera from immunized animals with peptide array was determined by ELISA. S1-S6, sera from swine immunized with RBD-based antigens. M1-M6, sera from mice immunized with RBD-based antigens. R300-R615, 20-mer overlapped peptides covering the RBD. The shade of blue is directly proportional to OD450 value. **(D–G)** Immunodominant epitope peptides binding with the antibodies in sera from swine/mouse immunized with RBD-based antigens. Closed circle, sera from swine immunized with RBD-based antigens. Closed square, sera from mice immunized with RBD-based antigens. Data was shown as mean ± SEM. *p < 0.05; **p < 0.01; ****p < 0.0001. Graphs were made in GraphPad Prism version 8.0.2.

To analyze the humoral response to SARS-CoV-2 RBD, 22 overlapping peptides with 5 amino acids offsets covering the RBD were synthesized based on the reference sequence of SARS-CoV-2 S protein (GenBank: YP_009724390) (**Figure 1A** and **Table 1**). To obtain higher mapping resolution, the truncation library was designed through a systematic truncation of the identified epitope peptides (**Table 4**). All peptides which were designed with cysteine residues at the N-terminus were synthesized by Sangon Biotech Co., Ltd. (Shanghai, China). The purity of the synthetic peptides was equal to or greater than 95%.

**Table 1:** Overlapped peptides spanning the RBD of SARS-CoV-2 S protein in this work.

Name	Peptides	Name	Peptides
R300	KCTLKSFTVEKGIYQTSNFR	R465	ERDISTEIYQAGSTPCNGVE
R315	TSNFRVQPTESIVRFPNITN	R480	CNGVEGFNCYFPLQSYGFQP
R330	PNITNLCPFGEVFNATRFAS	R495	YGFQPTNGVGYQPYRVVVLS
R345	TRFASVYAWNRKRISNCVAD	R510	VVVLSFELLHAPATVCGPKK
R360	NCVADYSVLYNSASFSTFKC	R525	CGPKKSTNLVKNKCVNFNFN
R375	STFKCYGVSPTKLNDLCFTN	R540	NFNFNGLTGTGVLTESNKKF
R390	LCFTNVYADSFVIRGDEVRQ	R555	SNKKFLPFQQFGRDIADTTD
R405	DEVRQIAPGQTGKIADYNYK	R570	ADTTDAVRDPQTLEILDITP
R420	DYNYKLPDDFTGCVIAWNSN	R585	LDITPCSFGGVSVITPGTNT
R435	AWNSNNLDSKVGGNYNYLYR	R600	PGTNTSNQVAVLYQDVNCTE
R450	NYLYRLFRKSNLKPFERDIS	R615	VNCTEVPVAIHADQL

### Peptide-Based ELISA

Peptide-based ELISA were performed according to described previously (33). Briefly, the 96-well plates were coated with the peptides (250 ng/well) in 0.05 M carbonate–bicarbonate buffer (CBS, pH 9.6) and incubated overnight at 4°C. After washing thrice with PBST (1× PBS with 0.05% Tween 20, pH 7.4), the plates were blocked with 5% skim milk at 37°C for 2h. Mouse serum samples diluted at 1:100 and swine serum samples diluted at 1:1000 were added to each well and incubated at 37°C for 30 min. The wells were washed thrice with PBST and incubated with HRP-conjugated goat anti-mouse IgG or goat anti-swine IgG diluted at 1:5000 at 37°C for 30 min. The reactions were developed using TMB. The OD values of each well were measured at 450 nm using an ELISA microplate reader.

### Monoclonal Antibodies Generation and Characterization

**Figure 2:**
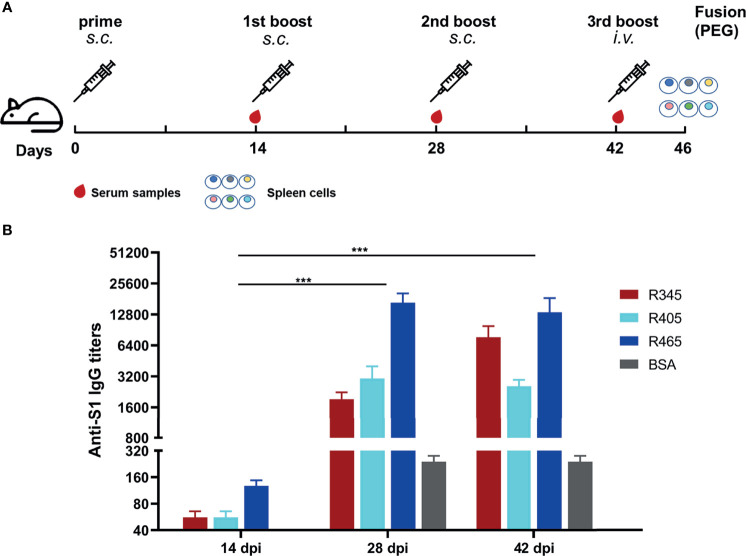
Immunization strategies and antibody responses in mice. **(A)** Scheme of immunization and sampling. Serum samples were collected 2 weeks after each immunization. Each immunogen plus with Freund’s adjuvant by subcutaneous (*s.c.*) injection for the immunizations at 0, 14, and 28 dpi. The mice with the highest antibody titers in each group were given the 3rd boost by intravenous (*i.v.*) injection at 42 dpi for mAbs preparation. **(B)** Titers of serum samples at 14, 28, and 42 dpi were detected by ELISA. Data was shown as mean ± SEM. ***p < 0.001. Graphs were made in GraphPad Prism version 8.0.2.

In order to prepare mAbs against these identified epitope peptides, the peptides (R345, R405, R465) designed with cysteine residues at the N-terminus were conjugated to the carrier protein, bovine serum albumin (BSA, Jackson ImmunoResearch Inc., USA), using the sulfosuccinimidyl 4-[N-maleimidomethyl] cyclohexane-1-carboxylate (Sulfo-SMCC, Thermo Scientific, USA). Peptide–BSA conjugates were served as the immunogens. The recombinant SARS-CoV-2 S1 protein (Sino Biological, Beijing, China) was used as the coating antigen in ELISA to determine serum titer of mice and screen for positive hybridomas. Twenty 6- to 8-week-old female BALB/c mice were randomly into four groups. The immunization scheme was as schematic **Figure 2A**. Mice were subcutaneously immunized with 20 μg of each immunogen emulsified with Freund’s adjuvant (Sigma-Aldrich, Shanghai, China) at 0, 14, and 28 days post prime-immunization (dpi). Mice from groups 1-3 were immunized with R345-BSA, R405-BSA, R465-BSA, respectively. Mice from group 4 were immunized with BSA as the negative control. All four groups were immunized three times at an interval of 2 weeks (0, 14, 28 dpi), while serum samples were collected 2 weeks after each immunization (14, 28, 42 dpi). The mice with the highest antibody titers in group 1-3 were given the last boost (at 42 dpi) with 40μg immunogens (without any adjuvant) by intravenous injection to prepare mAbs by hybridoma technology. Four days after the last boost (46 dpi), splenocytes from the mice were collected and fused with SP2/0 myeloma cells using PEG 1500. The unconjugated peptides (≥ 20 aa) could be used directly in the peptide-based ELISA (34). In order to obtain the specific mAbs against the B-cell epitope peptides, both the unconjugated peptides and SARS-CoV-2 S1 protein were used to screen for positive hybridomas, respectively. The positive hybridomas were further subcloned more than three times by the limiting dilution method. After subcloning, the positive hybridoma cells were injected into BALB/c mice and the ascites were collected. The subtypes of these mAbs were determined by mouse monoclonal antibody subtype identification kit (Proteintech, Wuhan, China). The antibody titers of these mAbs were detected by ELISA. Briefly, 96-well plates were coated with SARS-CoV-2 S1 protein. Hybridoma clone supernatants or ascites fluid were used as the primary antibody. The other steps refer to the peptide-based ELISA protocol. The neutralization capacity of these mAbs was assessed with a commercial SARS-CoV-2 Surrogate Virus Neutralization Test (sVNT) kit (GenScript, Nanjing, China) according to the manufacturer’s instruction. Negative control was a mAb against African swine fever virus p54 protein.

### Immunofluorescence Assay

The specific binding of these mAbs to SARS-CoV-2 S protein was further confirmed by IFA. Briefly, HEK293T cells were seeded at 2.5 × 10^5^ cells/well into a 24-well plate one day prior to transfection. When the cells in each well were 70–80% confluent, the cells were transfected with the recombinant plasmid pLVX-S that contained the full-length S protein gene (GenBank: YP_009724390). At 24 h post-transfection, the plates were fixed with methanol containing 1% H2O2 (precooled to −20°C) for 15 min at room temperature (RT). Then, the plates were washed with PBST and blocked with 5% skim milk. Next, the plates were incubated with the identified mAbs for 30 min at 37°C. Subsequently, the plates were washed for three times and incubated with goat anti-mouse IgG (H+L)-Alexa Fluor 488 (Invitrogen, Rockford, IL, USA). At last, the plates were stained by 4′,6-diamidino-2-phenylindole (DAPI, Solarbio, Beijing, China) and the fluorescence signals were developed by fluorescence microscopy (ZEISS, Jena, Germany).

### Western Blotting Analysis

The reactivity of these mAbs with the S proteins of SARS-CoV-2, SARS-CoV and MERS-CoV was determined by western blotting analysis. SARS-CoV-2 S1 subunit (YP_009724390.1, Val16-Arg685), SARS-CoV S1 subunit (AAX16192.1, Met1-Arg667) and MERS-CoV S1 subunit (AFS88936.1, Met1-Glu725) were purchased from Sino Biological Inc. The proteins were subjected to 10% NuPAGE™ Bis-Tris gels (Invitrogen, Rockford, IL, USA) and performed by blotting from the gels with iBlot™ 2 Transfer Stacks (Invitrogen, Rockford, IL, USA). The membranes were blocked with 5% skim milk and incubated with the identified mAbs, respectively. HRP-conjugated goat anti-mouse IgG was used with a 1:10000 dilution as the secondary antibody. The blots were exposed with enhanced chemiluminescent (ECL) substrate (NCM Biotech, China).

### Sequencing and Analysis of the mAb Variable Regions

The total RNA of hybridoma cell lines secreting the mAbs was isolated using TRIzol reagent (Invitrogen, USA) and the cDNA was synthesized using PrimeScript™ II 1st strand cDNA synthesis kit (Takara Biomedical Technology (Beijing) Co.) according to the manufacture’s protocols. The light- and heavy-chain (VL and VH) variable domains of the mAbs were amplified in two separate polymerase chain reaction (PCR) tests by two mouse Ig-Primer sets according to the previous methods (35, 36). The heavy chain amplification cycles were 95°C for 5 min; 35 cycles of 95°C for 30 sec, 55°C for 30 sec, 72°C for 1 min, and further extension at 72°C for 10 min. The light chain amplification reaction was similar to that of the heavy chain, except that the annealing temperature was replaced by 60°C. The PCR products were gel purified and sequenced by Sangon Biotech Co., Ltd (Shanghai, China). The VH and VL sequences were analyzed using IgBlast and IMGT/V-QUEST (37, 38). Based on results from the tools, the complementarity-determining regions (CDRs) were labeled. The amino acid sequences of the variable regions were aligned by ClustalW method. Tertiary structures of these mAbs were built using SWISS-MODEL and analyzed by the PyMOL Molecular Graphics System (Version 2.3.0, Schrödinger, LLC.).

### Conservation Analysis of the Identified Linear Epitopes

To assess the potential cross-reactive epitopes with its close relatives, the RBD sequence of SARS-CoV-2 (Wuhan-Hu-1) S protein was aligned with the consensus sequences from SARS-CoV (WH20) and MERS-CoV (HCoV-EMC). The multiple alignment was created with MegAlign. To deeply analyze the conservation of the identified linear epitopes in the currently circulating SARS-CoV-2 strains, all receptor binding site changes that occurred in different SARS-CoV-2 virus strains were extracted from GISAID database (https://www.gisaid.org/hcov19-mutation-dashboard/), where a total of 431,752 SARS-CoV-2 virus data was collected (updated on 2021-01-28).

## Results

### Analysis of B-Cell Epitope Peptides of Antibodies in the Sera From Animals Immunized With RBD-Based Antigens

The linear B-cell epitope peptides recognized by sera from animals immunized with RBD-based antigens were screened using 22 overlapping peptides with 5 amino acids offsets spanning the RBD of SARS-CoV-2 S protein (GenBank: YP_009724390) (**Figure 1A** and **Table 1**). Four linear B-cell epitope peptides (R345, R405, R450 and R465) of SARS-CoV-2 RBD were screened (**Figures 1B–G**). Three B-cell epitope peptides, R345, R405, and R465 strongly reacted with sera from some of the animals. The B-cell epitope peptide, R450, mildly reacted with all serum samples. Interestingly, two epitope peptides (R345, R405) were strongly recognized by swine sera, but weaker by mouse sera (**Figures 1D, E**). The epitope peptides (R450 and R465) were strongly recognized by mouse sera, but weaker by swine sera (**Figures 1F, G**). We speculated that this may be due to differences in ACE2 receptors between pigs and mice (39).

### Mouse Immunizations, Antibody Responses, and Generation of mAbs Targeting the Identified Linear B-Cell Epitope Peptides

The SARS-CoV-2 S protein-specific antibodies were detected by ELISA (**Figure 2B**). As shown in **Figure 2B**, the antibody titers at 28 dpi and 42 dpi were significantly higher than those at 14 dpi, indicating that potent antibody responses were induced. The antibody titer of group 1 (R345) at 42 dpi was slightly higher than that at 28 dpi, while the antibody titers of groups 2 (R405) and 3 (R465) at 42 dpi were slightly lower than their antibody titers at 28 dpi, indicating that the antibody titers had approximately reached the peak after three immunizations (**Figure 2B**). The mice with the highest antibody titers (12800 for R345, 3200 for R405, 25600 for R465) in each group were used as spleen donors for further mAbs preparation. The hybridoma cell lines stably secreting specific mAbs against the identified linear B-cell epitope peptides were screened by ELISA based on the peptides and SARS-CoV-2 S protein, and were named as 15G9 (anti-R345), 12C10 (anti-R405) and 10D2 (anti-R465). The antibody titers of these mAbs reached 409600, 819200 and 409600, respectively (**Table 2**). Subtype analysis revealed all mAbs are IgG1, and the light chain types are Kappa (**Table 2**).

**Table 2:** Characteristics of mAbs.

Mab name	Immunogen	Epitope type	Mab type	Titers		IFA	^‡^SVN
				Supernatants	Ascitic fluid		
15G9	R345-BSA	Linear	IgG1, Kappa	6400	409600	**^†^+**	**+**
12C10	R405-BSA	Linear	IgG1, Kappa	6400	819200	**+**	**+**
10D2	R465-BSA	Linear	IgG1, Kappa	800	409600	**+**	**+**

^†^Positive result.

^‡^Neutralization activity of the mAbs were assessed by a commercial SARS-CoV-2 surrogate virus neutralization test kit (Genscript, Nanjing, China).

### Binding and Neutralization Activity of These mAbs

**Figure 3:**
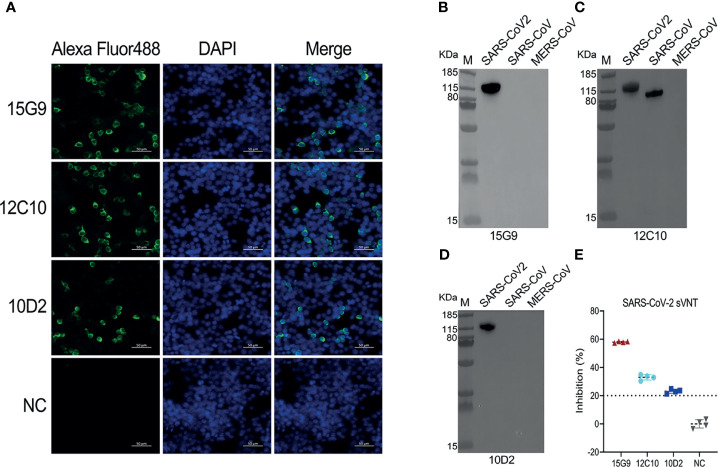
Characterization of the isolated mAbs (15G9, 12C10, 10D2). **(A)** Binding of the mAbs with SARS-CoV-2 S protein expressed in HEK293T cells. MAbs anti-SARS-CoV-2 S protein (green). Nuclei (blue). NC, Negative control. Scale bars, 50 μm. **(B–D)** The reactivity of mAbs with S1 proteins of SARS-CoV-2, SARS-CoV and MERS-CoV was measured by western blotting. **(E)** The neutralization capacity of the mAbs. NC, Negative control.

Binding analysis of the mAbs (15G9, 12C10 and 10D2) by IFA revealed that all three mAbs can specifically bind to the SARS-CoV-2 S protein expressed by HEK293T cells (**Figure 3A**). Results from western blotting showed that 15G9 and 10D2 reacted strongly with SARS-CoV-2 S protein, but did not react with SARS-CoV and MERS-CoV S proteins, indicating that the two mAbs specifically recognize SARS-CoV-2 S protein (**Figures 3B, D**). MAb 12C10 reacted strongly with the S proteins of both SARS-CoV-2 and SARS-CoV, indicating that the mAb is cross-reactive between the two coronaviruses, and implying that there may be a cross-reactive epitope in R405 (**Figure 3C**). The neutralization capacity of the mAbs was assessed by a commercial sVNT kit based on antibody-mediated blockage of ACE2-S protein interaction. The results of neutralization analysis showed that all three mAbs could inhibit the interaction between the SARS-CoV-2 S protein with the ACE2 receptor, indicating that they have neutralization activity (**Figure 3E** and **Table 2**).

### Detection of VH and VL of mAbs 15G9, 12C10 and 10D2

**Figure 4:**
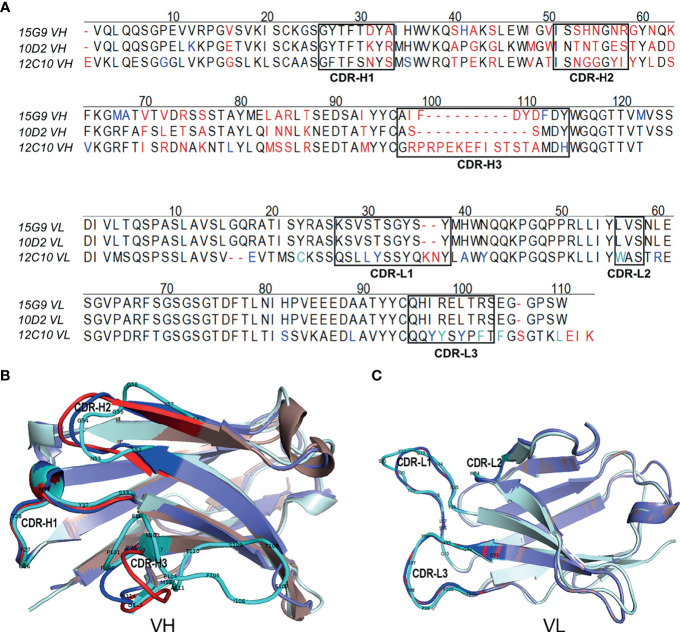
Sequence characteristics of the mAbs. **(A)** Multiple alignment of the variable regions of 15G9, 12C10 and 10D2. The black boxes indicated the complementarity-determining regions (CDRs). **(B)** Structural alignment of VHs for 15G9, 12C10 and 10D2. **(C)** Structural alignment of VLs for 15G9, 12C10 and 10D2. 15G9 (darksalmon), 12C10 (palecyan), 10D2 (slate). The CDRs of 15G9, 12C10, 10D2 were marked as red, cyan and blue, respectively. The variable region sequences of these mAbs had been submitted to GenBank and the accession numbers were MZ751046, MZ751047, MZ751048, MZ751049 and MZ751050, respectively.

To further characterize 15G9, 12C10 and 10D2, the variable region of each mAb was amplified and sequenced. Results from sequencing showed that each mAb only has one sequence, further confirming the monoclonality of the hybridoma cell lines (**Figure 4**). Sequence analysis revealed that 15G9 and 10D2 have the same VL, but the VHs have some difference. Mab 12C10 has vastly different V domains (VL and VH) with 15G9 and 10D2 (**Figure 4A**). Structural alignment further revealed that 15G9 and 10D2 have the identical VL and similar VH. But, 12C10 has distinct tertiary structures with 15G9 and 10D2, especially for CDR-H3 region (**Figures 4B, C**). By comparison with the published mouse sequences, the closest germline gene that the mAbs might originate from were identified, indicating that all three mAbs were derived from productively rearranged sequences. The closest germline genes encoded variable regions of these mAbs were shown in **Table 3**. The closest genes and alleles for the V and J gene of the kappa light chains are same in mAb 15G9 and mAb 10D2, while mAb 12C10 are different. Interestingly, the VL of 12C10 is minimally mutated with only one residue change from the germline gene, and the residue (Asn→Tyr) is located in the CDR-L1 region.

**Table 3:** Sequence characteristics of the SARS-CoV-2 mAbs.

Mab Name		CDRs			Closest germline gene and allele		
		CDR1	CDR2	CDR3	V	D	J
VH	15G9	GYTFTDYA	ISSHNGNR	AIFDYDFDY	IGHV1-67*01	IGHD2-4*01	IGHJ2*01
	10D2	GYTFTKYR	INTNTGES	ASSMDY	IGHV9-3*02	IGHD3-3*01	IGHJ4*01
	12C10	GFTFSNYS	ISNGGGYI	GRPRPEKEFISTSTAMDH	IGHV5-9-3*01	IGHD1-2*01	IGHJ4*01
VL	15G9	KSVSTSGYSY	LVS	QHIRELTRS	IGKV3-12*01	–	IGKJ2*01
	10D2	KSVSTSGYSY	LVS	QHIRELTRS	IGKV3-12*01	–	IGKJ2*01
	12C10	QSLLYSSYQKNY	WAS	QQYYSYPFT	IGKV8-30*01	–	IGKJ4*01

The number *01 signifies that any new polymorphic sequence will be described by comparison to that allele *01.

### Identification of Minimal Motifs of the Identified Epitope Peptides Using the mAbs

**Figure 5:**
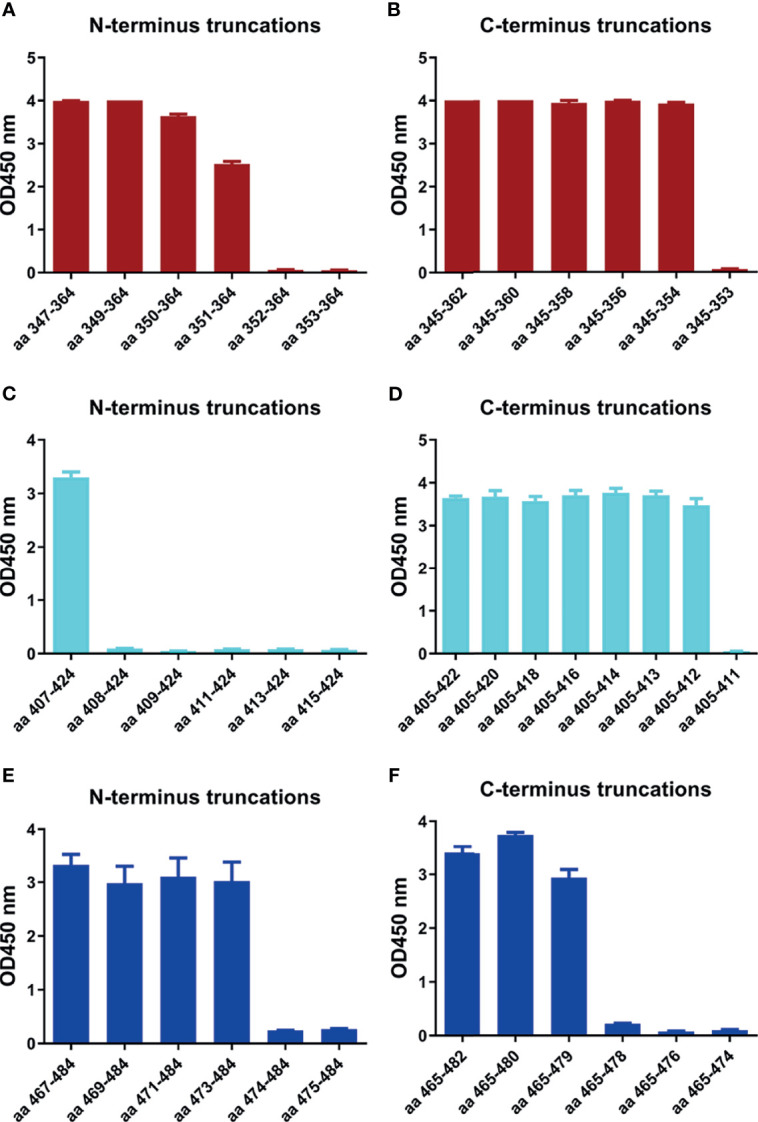
Minimal motifs recognized by the mAbs against the identified linear B-cell peptides. **(A, B)** N-terminal and C-terminal truncations of peptide R345. **(C, D)** N-terminal and C-terminal truncations of peptide R405. **(E, F)** N-terminal and C-terminal truncations of peptide R465.

In order to determine the minimal motif of the identified epitope peptides, the peptides (R345, R405, R465) were further truncated and characterized (**Table 4**). The results of peptide-based ELISA showed the reactivity of these truncated peptides with the corresponding mAbs (**Figure 5**). For peptide R345, the N-truncated peptide (^350^VYAWNRKRISNCVAD^364^) could be effectively bind to mAb 15G9, while the N-truncated peptide (^351^YAWNRKRISNCVAD^364^) had weaker reactivity, compared with peptide (^350^VYAWNRKRISNCVAD^364^). In addition, any peptide that was further truncated from the N-terminus of the peptide (^351^YAWNRKRISNCVAD^364^) could not bind to mAb 15G9 (**Figure 5A**). The C-truncated peptide (^345^TRFASVYAWN^354^) could be effectively recognized by mAb 15G9, while deletion any amino acids at the C-terminus of the peptide showed no reactivity (**Figure 5B**). These results suggested that the motif (^350^VYAWN^354^) is the minimal residues required for antibody recognition. For R405, the N-truncated peptide (^407^VRQIAPGQTGKIADYNYK^424^) could be well recognized by mAb 12C10, but the binding capability of truncated peptides was completely lost when ^407^Val was deleted (**Figure 5C**). The C-truncated peptides of R405 were shown to bind mAb 12C10 strongly until ^412^Gln was removed (**Figure 5D**). This indicated that the motif ^407^VRQIAP^412^ is a precise linear epitope for antibody binding. MAb 10D2 specific to R465 effectively recognized the N-truncated peptides until the deletion of ^473^Tyr, while the C-truncated peptides shown to bind mAb 10D2 strongly until ^479^Pro was removed, indicating that the linear B-cell epitope in R465 is ^473^YQAGSTP^479^ and both of ^473^Tyr and ^479^Pro are critical residues for epitope- antibody interaction (**Figures 5E, F**).

**Table 4:** Truncation library of the identified epitope peptides used in this work.

Name	Location (aa)	N-term truncations	Name	Location (aa)	C-term truncations
**R345-N2**	**347-364**	**CFASVYAWNRKRISNCVAD**	**R345-C2**	**345-362**	**CTRFASVYAWNRKRISNCV**
**R345-N4**	**349-364**	**CSVYAWNRKRISNCVAD**	**R345-C4**	**345-360**	**CTRFASVYAWNRKRISN**
**R345-N6**	**351-364**	**CYAWNRKRISNCVAD**	**R345-C6**	**345-358**	**CTRFASVYAWNRKRI**
R345-N8	353-364	CWNRKRISNCVAD	**R345-C8**	**345-356**	**CTRFASVYAWNRK**
R345-N10	355-364	CRKRISNCVAD	**R345-C10**	**345-354**	**CTRFASVYAWN**
**R405-N2**	**407-424**	**CVRQIAPGQTGKIADYNYK**	**R405-C2**	**405-422**	**CDEVRQIAPGQTGKIADYN**
R405-N4	409-424	CQIAPGQTGKIADYNYK	**R405-C4**	**405-420**	**CDEVRQIAPGQTGKIAD**
R405-N6	411-424	CAPGQTGKIADYNYK	**R405-C6**	**405-418**	**CDEVRQIAPGQTGKI**
R405-N8	413-424	CGQTGKIADYNYK	**R405-C8**	**405-416**	**CDEVRQIAPGQTG**
R405-N10	415-424	CTGKIADYNYK	**R405-C10**	**405-414**	**CDEVRQIAPGQ**
**R465-N2**	**467-484**	**CDISTEIYQAGSTPCNGVE**	**R465-C2**	**465-482**	**CERDISTEIYQAGSTPCNG**
**R465-N4**	**469-484**	**CSTEIYQAGSTPCNGVE**	**R465-C4**	**465-480**	**CERDISTEIYQAGSTPC**
**R465-N6**	**471-484**	**CEIYQAGSTPCNGVE**	R465-C6	465-478	CERDISTEIYQAGST
**R465-N8**	**473-484**	**CYQAGSTPCNGVE**	R465-C8	465-476	CERDISTEIYQAG
R465-N10	475-484	CAGSTPCNGVE	R465-C10	465-474	CERDISTEIYQ
**R345-N5**	**350-364**	**CVYAWNRKRISNCVAD**	R345-C11	345-353	CTRFASVYAW
R345-N7	352-364	CAWNRKRISNCVAD	**R405-C11**	**405-413**	**CDEVRQIAPG**
R405-N3	408-424	CRQIAPGQTGKIADYNYK	**R405-C12**	**405-412**	**CDEVRQIAP**
R465-N9	474-484	CQAGSTPCNGVE	R405-C13	405-411	CDEVRQIA
			**R465-C5**	**465-479**	**CERDISTEIYQAGSTP**

The truncated peptides that reacted with the corresponding mAbs.

### The Conservation of the Identified Linear B-Cell Epitopes

**Figure 6:**
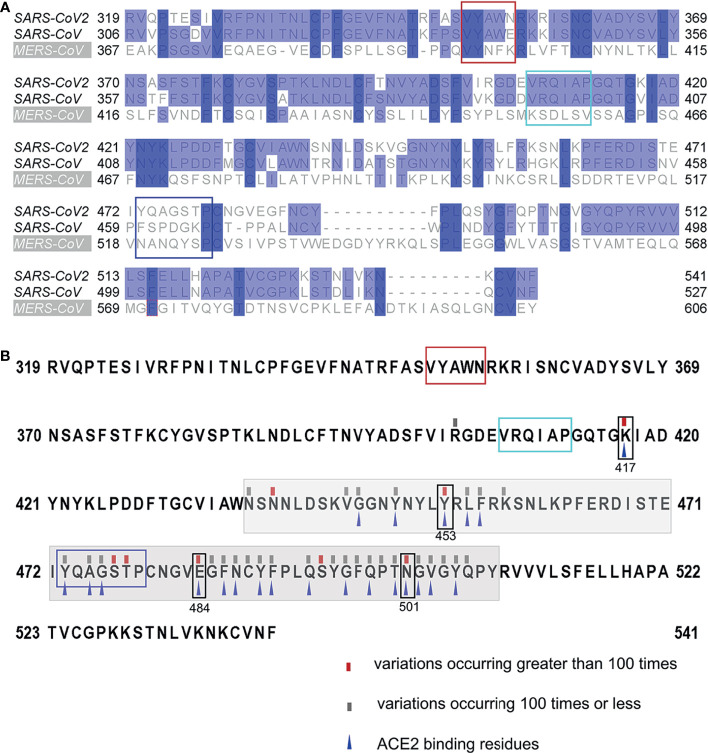
Conservation of the identified linear B-Cell epitopes. **(A)** Multiple sequence alignments of the RBDs of SARS-CoV-2 (Wuhan-Hu-1), SARS-CoV (WH20) and MERS-CoV (HCoV-EMC). **(B)** Mutations found in the RBDs of the currently circulating SARS-CoV-2 strains. All receptor binding site changes reported in GISAID (updated on 2021-01-28) were listed in the figure. The red rectangles indicated variations occurring greater than 100 times at the site. The grey rectangles indicated variations occurring 100 times or less at the site. The grey shading indicated the sequence of the RBM. The blue triangles indicated the residues that interact with ACE2. The box indicated the identified epitopes, epitope ^351^YAWN^354^ (red), epitope ^407^VRQIAP^412^ (cyan), epitope ^473^YQAGSTP^479^ (blue). The black boxes indicated the mutation sites in RBD, included K417N, E484K and N501Y of the novel variant 501Y.V2, N501Y of the B.1.1.7 variant, and Y453F of mink-associated variant strains.

All residues of epitope ^407^VRQIAP^412^ were conserved between SARS-CoV-2 and SARS-CoV, explaining the cross-reactivity of mAb 12C10 and indicating that epitope ^407^VRQIAP^412^ is a cross-reactive epitope for SARS-CoV and SARS-CoV-2 (**Figure 3C** and **Figure 6A**). Out of 5 residues in the epitope ^350^VYAWN^354^ of SARS-CoV-2, four were conserved between SARS-CoV-2 and SARS-CoV, only one substitution (^341^E→^354^N) (**Figure 6A**). Nonetheless, mAb 15G9 targeting the linear epitope ^350^VYAWN^354^ reacted with SARS-CoV-2 S1, but not with SARS-CoV S1 protein (**Figure 3B**). The results indicated that the difference in reactivity of mAb 15G9 between SARS-CoV-2 and SARS-CoV S proteins was likely due to the residue change (^341^E→^354^N). The sequence of epitope ^473^YQAGSTP^479^ located in RBM, a critical region for ACE2-binding, was vastly different to SARS-CoV and MERS-CoV, revealing the molecular basis of mAb 10D2 specific binding to SARS-CoV-2 (**Figure 3D** and **Figure 6A**). To deeply analyze the conservation of the identified linear epitopes in the currently circulating SARS-CoV-2 strains, all receptor binding site changes obtained from GISAID database which contained a total of 431,752 virus data (updated on 2021-01-28) were labeled (**Figure 6B**). It could be seen that epitope ^350^VYAWN^354^ and epitope ^407^VRQIAP^412^ were highly conserved among different SARS-CoV-2 strains. Epitope ^473^YQAGSTP^479^ overlapped with ACE2-binding residues (^473^Y, ^475^A and ^476^G) was variable, especially for residues ^477^S and ^478^T. Currently, ^477^S had 7 forms of mutations: S477N, S477R, S477I, S477G, S477N, S477T. S477K and S477N was the dominant mutation with a frequency of 21465, second only to N501Y, which has raised public concerns. There were four mutations at site 478: T478I, T478K, T478R, T478A, and the frequency of each mutation was 218, 69, 30, 2, respectively.

### Structural Analysis of the Identified Linear B-Cell Epitopes

**Figure 7:**
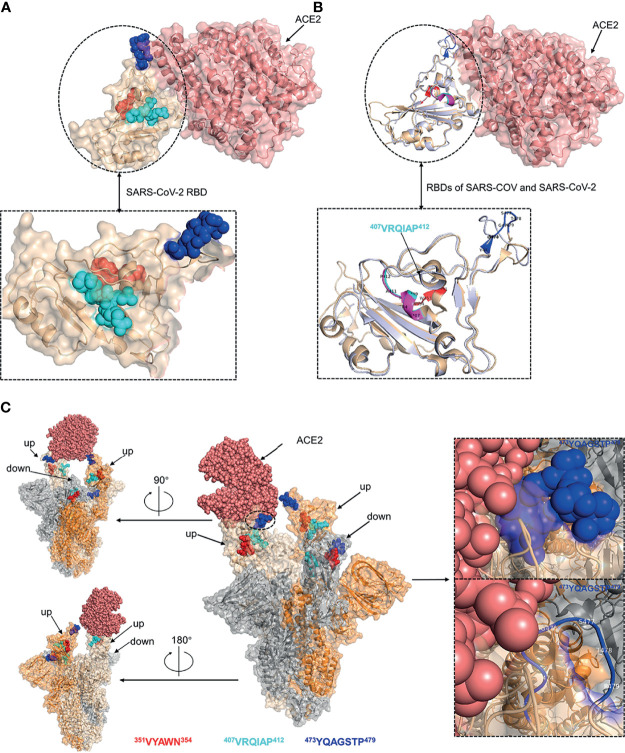
Structural analysis of the identified linear B-cell epitopes. **(A)** The linear B-cell epitopes were mapped on the structure of SARS-CoV-2 RBD-ACE2 complex (PDB ID: 6M0J). Inset was a zoomed-in view of the SARS-CoV-2 RBD. **(B)** The structure of SARS-CoV RBD-ACE2 complex (PDB ID: 2AJF) was superimposed with the structure of SARS-CoV-2 RBD-ACE2 complex (PDB ID: 6M0J). Cartoon representation of the RBDs of SARS-CoV and SARS-CoV-2. Inset was a zoomed-in view of the RBDs of SARS-CoV and SARS-CoV-2. **(C)** The localization of identified epitopes mapped on SARS-CoV-2 S trimer (PDB ID: 7A95). Two RBDs in the open state and one RBD in the closed state. The S monomer configured ‘up’ and complexed with ACE2 was colored as wheat. The other ‘up’ configured S monomer was colored as orange. The S monomer in the closed state was colored as grey. Insets were zoomed-in views of the localization of epitope ^473^YQAGSTP^479^. Throughout the whole figure, SARS-CoV RBD (whiteblue), SARS-CoV-2 RBD (wheat), ACE2 (salmon pink), epitope ^407^VRQIAP^412^ on SARS-CoV RBD (magentas), epitope ^407^VRQIAP^412^ on SARS-CoV-2 RBD (cyan), epitope ^350^VYAWN^354^ (red), epitope ^473^YQAGSTP^479^ (blue).

The spatial distribution and structural property of the experimentally identified epitopes were analyzed by mapping to the SARS-CoV-2 RBD-ACE2 complex (PDB ID: 6M0J) and S trimer (PDB ID: 7A95). Epitope ^350^VYAWN^354^ and epitope ^407^VRQIAP^412^ were located in distinct face of SARS-CoV-2 RBD, and epitope ^473^YQAGSTP^479^ was located at the RBD loop that bound with the ACE2 receptor (**Figure 7A**). The superposed structure of SARS-CoV RBD-ACE2 complex (PDB ID: 2AJF) and SARS-CoV-2 RBD-ACE2 complex (PDB ID: 6M0J) showed that all the three epitopes contained loop region and epitope ^407^VRQIAP^412^ was identical between SARS-CoV and SARS-CoV-2, implying that the three epitopes were easily accessible to the antibodies and further confirming that epitope ^407^VRQIAP^412^ was a common epitope of SARS-CoV and SARS-CoV-2 (**Figure 7B**). Like other coronaviruses, SARS-CoV-2 RBD switches between “up” and “down” conformations by hinge-like movements and can interact with ACE2 only when it is in the “up” configuration (10, 40). Epitope ^350^VYAWN^354^ located at the exposed surface of S trimer, implying it was accessible to the antibody in both the “down” and “up” forms of S protein (**Figure 7C**). Interestingly, the cross-reactive epitope (^407^VRQIAP^412^) of SARS-CoV and SARS-CoV-2 was buried and inaccessible to the antibody when the RBD existed in a “down” conformation (**Figure 7C**). Epitope ^473^YQAGSTP^479^ located in RBM region of the spike head and overlapped with the ACE2-binding sites, further showing that it was a potential target for stimulating neutralizing antibody that interfere with virus–receptor interactions (**Figure 7C**).

## Discussion

The ongoing COVID-19 pandemic caused by SARS-CoV-2 poses a huge threat to global public health and is disrupting societies and economies worldwide. The RBD of SARS-CoV-2 S protein plays an essential role in viral infection and is considered as a major antigen for vaccine design (41, 42). Understanding the humoral response to the RBD of SARS-CoV-2 S protein may help find more targeted biomarkers for COVID-19 detect and vaccine development. In this study, the humoral response to RBD-based antigen was profiled by screening the linear B-cell epitopes in a panel of sera from animals (swine/mouse) immunized with RBD-based antigens. Mice are the most commonly used animals in the laboratory for preliminary evaluation of antigen. However, the previous reports had shown that ACE2, the receptor for SARS-CoV-2, from human, *Rhinolophus sinicus* (bat), civet, swine but not mouse mediate SARS-CoV-2 infection *in vitro* (39, 43). Therefore, the swine was also chosen for immunization and evaluation. Furthermore, mAbs (15G9, 12C10, 10D2) binding to the linear B-cell peptides were generated and characterized, and three immunodominant linear B-cell epitopes, ^350^VYAWN^354^, ^407^VRQIAP^412^ and ^473^YQAGSTP^479^, on the RBD of SARS-CoV-2 S protein, were finely mapped using the mAbs. These findings may facilitate further understanding the antigenic structure in the SARS-CoV-2 RBD and development of vaccines and immune-based diagnosis.

Analysis of serum antibodies induced by RBD-based antigens revealed four linear antigenic targets (R345, R405, R450 and R465) (**Figure 1**). Previous reports had identified that the peptides S456-460 and S455-469 containing an identical linear B-cell epitope overlap with ACE2-binding residues (31, 44, 45). In addition, the epitope partially overlaps the binding sites of the neutralizing antibody CB6 and the antibody induced by S455-469 had a neutralizing effect on the pseudovirus of SARS-CoV-2, suggesting that it is a neutralizing epitope (44, 45).Consistent with the previous reports, R450 (S450-469) includes the same linear B-Cell epitope with the peptides S456-460 and S455-469, indicating that it is a potential neutralizing epitope peptides and can be used as a SARS-CoV-2 vaccine candidate. Peptide immunization *in vivo* showed that the peptides (R345, R405, R465) could induce strong and specific immune responses to SARS-CoV-2 S protein, confirming that they are linear B-cell epitope peptides of SARS-CoV-2 S protein (**Figure 2B**). Furthermore, three hybridoma cell lines secreting the mAbs (15G9, 12C10 and 10D2) that targeted the peptides (R345, R405 and R465) were generated and characterized, respectively (**Figure 3** and **Table 2**). Both of 15G9 and 10D2 specifically recognized the S protein of SARS-CoV-2 (**Figures 3B, D**), whereas mAb12C10 could strongly bind to both of SARS-CoV-2 and SARS-CoV S proteins (**Figure 3C**), indicating that 12C10 is a cross-reactive antibody between SARS-CoV-2 and SARS-CoV. Neutralization analysis showed that all three mAbs had neutralization activity. Consistent with a previous report, mAb 12C10 of R405 and mAb 10D2 of R465 inhibited the RBD-ACE2 interaction with an inhibition rate of 20%–40% (**Figure 3E**), suggesting that R405 and R465 were able to elicit neutralizing antibodies (45).

The amino acid sequence of an antibody, especially the CDRs, is the core of its biological function, and responsible for antibody-antigen response (46, 47). Hybridoma cell lines secreting mAbs may be lost or mutated due to storage accidents, gene drift, or contamination. To protect and characterize the mAbs, we further sequenced and analyzed the variable regions of these antibodies (15 G9, 12C10 and 10D2). Only one sequence was found for each mAb, further confirming the monoclonality of the hybridoma cell lines. Sequence analysis revealed that all three mAbs were derived from productively rearranged sequences. The CDRs of heavy chain and kappa light chain were also characteristically annotated (**Figure 4** and **Table 3**). A recent study reported that the average CDR-H3 length of SARS-CoV-2 mAb was longer compared to the IgG repertoires of three healthy human donors, consistent with our study (48). The previous report also showed that the predominant subtype of mAbs against SARS-CoV-2 spike is IG1, while all the three mAbs are IgG1 in our work (**Table 2**). In addition, the sequencing analysis of these mAbs may facilitate further antibody engineering, such as species, isotype and subtype switching, and antibody humanization by methods of speciation and affinity maturation.

In order to determine the minimal binding motif of the mAbs (15G9, 12C10 and 10D2), the peptides (R345, R405, R465) were further truncated. As shown in **Figure 5**, ^350^VYAWN^354^, ^407^VRQIAP^412^ and ^473^YQAGSTP^479^ are the precise epitopes for mAbs binding. Epitope ^350^VYAWN^354^ overlaps with epitope S348-357 identified by serological analysis of COVID-19 patients, suggesting that it is a natural linear epitope and can be used as a candidate for COVID-19 diagnosis (44). Up to now, no mutation located in epitope ^350^VYAWN^354^ had been found in a total of 431,752 SARS-CoV-2 virus strains, indicating that it was highly conserved among different SARS-CoV-2 strains (**Figure 6B**). Epitope ^407^VRQIAP^412^ is consistent with some identified neutralizing epitopes, such as S406-415, S406–420 and S404-426 (44, 45, 49). MAb 12C10 binding to epitope ^407^VRQIAP^412^ had cross-reactivity between SARS-CoV-2 and SARS-CoV, implying that epitope ^407^VRQIAP^412^ is a cross-reactive epitope for SARS-CoV-2 and SARS-CoV. Further alignment analysis of SARS-CoV-2 (Wuhan-Hu-1), SARS-CoV (WH20) and MERS-CoV (HCoV-EMC) validated that ^407^VRQIAP^412^ is the common epitope of SARS-CoV-2 and SARS-Co-V, explaining the mechanism of mAb 12C10 cross-reaction between SARS-CoV-2 and SARS-CoV (**Figure 6A**). Interesting, epitope ^407^VRQIAP^412^ overlaps with the epitope of a potent therapeutic antibody, H104, that efficiently neutralized SARS-CoV-2 and SARS-CoV pseudoviruses as well as authentic SARS-CoV-2, suggesting that ^407^VRQIAP^412^ would also a common neutralizing epitope of SARS-CoV-2 and SARS-CoV (25). In addition, ^407^VRQIAP^412^ was highly conserved across various virus isolates. Therefore, it was considered to be a promising candidate for structure-based universal vaccine design. Like CR3022, epitope ^407^VRQIAP^412^ was located in the trimeric interface and was only exposed on the “up” conformation (**Figure 7C**), implying mAb 12C10 would sterically block ACE2 receptor binding (21). Epitope ^473^YQAGSTP^479^ also partially overlaps the binding sites of the human neutralizing mAb CB6, indicating that the epitope is a neutralizing epitope (13). Similar to CB6, it may be inferred that the antibody targeting epitope ^473^YQAGSTP^479^ may interfere with virus–receptor interactions through both steric hindrance and direct competition with interface residues. By comparing with the mutations documented in 431,752 SARS-CoV-2 strains (GISAID), we found that five (^473^Y, ^475^A,^476^G, ^477^S and ^478^T) out of 7 residues in epitope ^473^YQAGSTP^479^ were variable (**Figure 6B**). Epitope ^473^YQAGSTP^479^ is localized in RBM and the residues 473, 475 and 476 are ACE2-binding sites (**Figure 7**). Some novel SARS-CoV-2 variants has been found to harbor mutations in the S protein, and increase the affinity between SARS-CoV-2 and the ACE2 receptor, accelerate the transmission of the virus and exhibit substantial or complete escape from therapeutically relevant mAbs/convalescent plasma (https://virological.org/t/preliminary-genomic-characterisation-of-an-emergent-sars-cov-2-lineage-in-the-uk-defined-by-a-novel-set-of-spike-mutations/563) (50–52). Most neutralizing epitopes in the RBD of SARS-CoV S protein could be completely disrupted by single amino acid substitutions (e.g., D429A, R441A or D454A) or by deletions of several amino acids at the N-terminal or C-terminal region of the RBD (53). Although the mutations at sites 473, 475 and 476 presented in a few virus strains at present, but they should still cause a great concern of the potential to reduce the binding affinity and effectiveness of antibodies. Currently, no evidence showed that the mutations at sites 477 and 478 of SARS-CoV-2 S protein result in reducing the protection of antibodies and affecting virus-host interaction. However, the mutations occurred in epitope ^473^YQAGSTP^479^, which located in RBM and overlapped with ACE2-binding residues, probably beneficial for the virus in some way that has not been revealed. In addition, the mutations frequency of site 477 was 21465, second only to N501Y (with a frequency of 26174). It may be also beneficial for the virus when the same mutation is independently selected multiple times. Actual effects of these mutations will require further efforts.

Overall, four linear B-cell epitope peptides of SARS-CoV-2 (R345, R405, R450 and R465) were screened utilizing sera from animals vaccinated with RBD-based antigens and strong responses to three linear B-cell epitope peptides (R345, R405 and R465) were observed. The immunogenicity of the three peptides was further accessed by peptide immunization in mice and all of them could induced potent antibody response to SARS-CoV-2 S protein. Furthermore, three potential neutralizing mAbs (15G9, 12C10 and 10D2) binding to the antigenic peptides (R345, R405, R465) were further generated and characterized. Among of these antibodies, 12C10 is a cross-reactive antibody against SARS-CoV-2 and SARS-CoV. In addition, the variable regions of these antibodies were sequenced and three immunodominant epitopes in the SARS-CoV-2 RBD were finely mapped using these mAbs. Among of these epitopes, ^350^VYAWN^354^ is specific for SARS-CoV-2 S protein and highly conserved in different SARS-CoV-2 strains; ^407^VRQIAP^412^ is a cross-reactive epitope shared between SARS-CoV-2 and SARS-CoV; ^473^YQAGSTP^479^ located in RBM is variable among different SARS-CoV-2 strains. Aside from scientific significance for understanding the antigenic structure, function, antibody–antigen interaction, these findings may facilitate further efforts to design SARS-CoV-2 vaccines and the target of COVID-19 diagnostic.

## Data Availability Statement

The original contributions presented in the study are included in the article/supplementary material. Further inquiries can be directed to the corresponding author.

## Ethics Statement

The animal study was reviewed and approved by the Ethical and Animal Welfare Committee of Henan Academy of Agricultural Sciences (Approval number SYXK 2021-0003).

## Author Contributions

GZ, AW and MJ conceptualized and designed this study. MJ, PD, YT and YW conducted the most experiments. YL collected and prepared the serum samples. MJ and HL performed figure preparation and prepared the manuscript. AW revised the manuscript. All authors contributed to the article and approved the submitted version.

## Funding

This work was supported by the National Natural Science Foundation of China (Grant No. 32072944); Henan Scientific Research Project on Prevention and Control of COVID-19 epidemic (Grant No. 211100310200).

## Conflict of Interest

HL, YL, YT and YW were employed by Henan Zhongze Bioengineering Co., Ltd.

The remaining authors declare that the research was conducted in the absence of any commercial or financial relationships that could be construed as a potential conflict of interest.

## Publisher’s Note

All claims expressed in this article are solely those of the authors and do not necessarily represent those of their affiliated organizations, or those of the publisher, the editors and the reviewers. Any product that may be evaluated in this article, or claim that may be made by its manufacturer, is not guaranteed or endorsed by the publisher.
